# RETHINKING NURSING HOME ARCHITECTURE AND DESIGN IN THE LIGHT OF THE COVID-19 PANDEMIC

**DOI:** 10.1093/geroni/igac059.2855

**Published:** 2022-12-20

**Authors:** Desmond O’Neill, Tom Grey, Dimitra Xidous, Jennifer O’Donoghue, Mehak Puntambekar

**Affiliations:** Trinity College Dublin, Dublin, Dublin, Ireland; Trinity College Dublin, Dublin, Dublin, Ireland; Trinity College Dublin, Dublin, Dublin, Ireland; Trinity College Dublin, Dublin, Dublin, Ireland; Trinity College Dublin, Dublin, Dublin, Ireland

## Abstract

**Introduction:**

The huge death rate in nursing homes during the COVID-19 pandemic raised serious questions as to whether the built environment of nursing homes was a factor in this very high mortality, as well as a factor in quality of life.

**Method:**

We embarked on a wide-ranging study involving a review of Irish policy, stakeholder engagement, Irish case studies, literature review, and international case studies to understand the key issues that influence the planning, design, and operation of nursing home settings settings, and to identify how these shape care models and the physical environment.

**Results:**

The project generated the following key themes: a) including the voices of residents, family and staff in co-creation of design and research; b) integrating nursing homes with the overall housing spectrum; c) linking nursing homes with ageing in place policy; d) further research on optimal design; e) understanding resident diversity; f) greater inclusion of Universal Design principles; g) designing for resilience; and h) Convergence between infection control and quality of life Discussion: Our Research Findings have been developed to identify major current issues related to the built environment and its role in creating a balance between quality of life and COVID-19 infection control in Irish and international nursing home settings. These findings are relevant for a wide range of stakeholders and will be disseminated across a number of channels to continue this conversation and help to continue the evolution of nursing home design.

